# Pseudocapacitive Effects of Multi-Walled Carbon Nanotubes-Functionalised Spinel Copper Manganese Oxide

**DOI:** 10.3390/nano12193514

**Published:** 2022-10-08

**Authors:** Christopher Nolly, Chinwe O. Ikpo, Miranda M. Ndipingwi, Precious Ekwere, Emmanuel I. Iwuoha

**Affiliations:** Sensor Laboratories (SensorLab), Department of Chemistry, University of the Western Cape, Robert Sobukwe Road, Bellville, Cape Town 7535, South Africa

**Keywords:** galvanostatic charge/discharge, nanocomposite electrode, pseudocapacitor, specific capacitance, spinel metal oxide

## Abstract

Spinel copper manganese oxide nanoparticles combined with acid-treated multi-walled carbon nanotubes (CuMn_2_O_4_/MWCNTs) were used in the development of electrodes for pseudocapacitor applications. The CuMn_2_O_4_/MWCNTs preparation involved initial synthesis of Mn_3_O_4_ and CuMn_2_O_4_ precursors followed by an energy efficient reflux growth method for the CuMn_2_O_4_/MWCNTs. The CuMn_2_O_4_/MWCNTs in a three-electrode cell assembly and in 3 M LiOH aqueous electrolyte exhibited a specific capacitance of 1652.91 F g^−1^ at 0.5 A g^−1^ current load. Similar investigation in 3 M KOH aqueous electrolyte delivered a specific capacitance of 653.41 F g^−1^ at 0.5 A g^−1^ current load. Stability studies showed that after 6000 cycles, the CuMn_2_O_4_/MWCNTs electrode exhibited a higher capacitance retention (88%) in LiOH than in KOH (64%). The higher capacitance retention and cycling stability with a Coulombic efficiency of 99.6% observed in the LiOH is an indication of a better charge storage behaviour in this electrolyte than in the KOH electrolyte with a Coulombic efficiency of 97.3%. This superior performance in the LiOH electrolyte than in the KOH electrolyte is attributed to an intercalation/de-intercalation mechanism which occurs more easily in the LiOH electrolyte than in the KOH electrolyte.

## 1. Introduction

The rapid increase in energy demand and consumption of fossil fuel resources has evoked the need for developing renewable and self-sustaining energy generation systems, such as solar cells, wind and hydroelectric turbines [1]. However, due to the intermittent nature of solar and wind energy, including the expensive construction cost of hydropower plants, high performance energy storage technologies are in great demand [2]. In this regard, supercapacitors possess unique advantages, such as high specific power, extended cycle life, fast charge/discharge rates, light weight and environmental benignity [3]. However, the major limitation of supercapacitors for practical applications is their low specific energy. This therefore provides motive to develop supercapacitor electrode materials capable of generating enhanced energy storage capacities without jeopardizing their high specific power and cycling stability.

Supercapacitors are classified as electric double-layer capacitors (EDLCs) and pseudocapacitors (PCs) according to their specific charge storage mechanism. EDLCs involve the use of carbon-based materials such as activated carbon, carbon nanotubes and grapheme as electrode materials to store charges by electrostatic charge adsorption and desorption processes, whereas PCs use metal oxide/hydroxide and conducting polymer electrode materials to store charges via fast and reversible faradaic redox reactions throughout the material surface [4]. Recently, numerous transition metal oxides (TMOs) such as CuO, MnO_2_, Mn_3_O_4_, RuO_2_, ZnO, NiO and Co_3_O_4_ have been researched as electrode materials for supercapacitors [5,6,7,8,9,10,11]. Wang et al. reported the performance of Mn_3_O_4_ nanomaterials synthesized via a two-step hydrothermal method and applied in asymmetric supercapacitors [12]. Another approach to supercapacitors development is to integrate manganese oxides with other transition metals such as Ni, Zn and Co in formulating spinel structured NiMn_2_O_4_, ZnMn_2_O_4_ and CoMn_2_O_4_ nanomaterials [13,14,15,16]. Saravanakumar et al. synthesized CuMn_2_O_4_ nanoparticles with rice-like morphology, through a solvothermal method for pseudocapacitor applications [17]. However, manganese oxide-based electrodes suffer from low-rate capabilities and poor cycling stability due to crystallographic defects and heavy aggregation of particles which render fewer number of electro-active sites for diffusion of electrolyte ions [18]. A strategic approach to overcome this challenge is by designing a nanocomposite of the manganese oxide electrode with carbon-based materials for increased surface area, electro-activity and cycling stability.

Carbon nanotubes (CNTs), due to their unique properties such as excellent electrical conductivity, good thermal stability, high flexibility, high specific surface area (50–1315 m^2^ g^−1^) and high surface area-to-volume ratio, have been employed in the development of nanocomposite electrodes for supercapacitors because of their ability to enhance the specific capacitance, energy and power density of the materials [19,20]. Shakir et al. demonstrated a facile two-step fabrication method for synthesizing spinel nickel cobaltite (NiCo_2_O_4_) nanostructures anchored to MWCNTs for flexible pseudocapacitors [21]. In another investigation, Geng et al. fabricated NiCo_2_O_4_/CNT core-shell nanocomposite hybrids by in-situ synthesis of ultrafine NiCo_2_O_4_ nanoparticles onto acid-functionalized CNT-films [22]. The regulation effects of CNT pre-treatment were mainly investigated on the electrochemical characteristics of NiCo_2_O_4_/CNT nanocomposites as electrode materials for flexible pseudo-capacitors. The study concluded that the introduction of redox-active heteroatoms such as carbon, oxygen, hydrogen, nitrogen and sulphur onto the MWCNTs network promoted a pseudocapacitive mechanism in conjunction with the EDLC mechanism.

Composite electrode materials such as ZnMn_2_O_4_/MWCNTs, Mn_3_O_4_/MWCNTs, V_2_O_5_/CNT and MnCoO_x_/MWCNTs [23,24,25,26,27,28] have also been reported. Fabricating metal oxides with MWCNTs promotes a dual charge storage mechanism comprised of an electrochemical double layer capacitor (EDLC) mechanism from MWCNTs and the Faradaic redox reaction mechanism. The MWCNTs support structure additionally provides good structural flexibility, high aspect ratio and strong mechanical strength which helps to reduce nanoparticle agglomeration [26]. Although CuMn_2_O_4_ has been synthesized for supercapacitor applications [16], CuMn_2_O_4_/MWCNTs electrode material has not been reported for supercapacitors.

Leveraging the unique properties of MWCNTs, CuMn_2_O_4_/MWCNTs nanocomposite, in addition to the pristine electrode materials, was synthesized in this work, and the electrochemical properties of the materials investigated in two different aqueous electrolytes (3 M KOH and 3 M LiOH) in order to ascertain the electrolyte where the electrode demonstrated better cycling stability, since electrochemical stability is related to the cycle life and safety of supercapacitors [29]. The distribution of CuMn_2_O_4_ nanoparticles throughout the MWCNTs framework was made flexible by first introducing hydrophilic groups to the MWCNTs surface through functionalization in H_2_SO_4_-HNO_3_ acid mixture. These groups function as nucleation sites for in situ positioning of CuMn_2_O_4_ nanoparticles on the MWCNTs surface. The functionalised nanotubes provided good mechanical support, increased surface area and more efficient electronic channels for faster ion diffusion as evidenced in the improved structural and electrochemical stability of the CuMn_2_O_4_/MWCNTs nanocomposite.

## 2. Experimental Section and Characterizations

### 2.1. Materials and Reagents

The list of chemical reagents used in all electrode material synthetic procedures include potassium permanganate (KMnO_4_, 99.0%), diethylene glycol (C_4_H_10_O_3_, 99.0%), ethanol (CH_3_CH_2_OH, 99.8%), copper (II) nitrate trihydrate (Cu(NO_3_)_2_·3H_2_O, 99%), manganese (II) nitrate tetrahydrate (Mn(NO_3_)_2_·4H_2_O, 99%), nitric acid (HNO_3_, 65%), citric acid monohydrate (C_6_H_8_O_7_·H_2_O, 99.5%), ammonium hydroxide (NH_4_OH, 25%), raw multi-walled carbon nanotubes (MWCNTs, 95%), sulphuric acid (H_2_SO_4_, 98%), polyvinylpyrrolidone (PVP, average mol wt 40,000), hydrazine hydrate (N_2_H_4_·H_2_O, 50–60%), carbon black (CB), polyvinylidene fluoride (PVDF), N-methyl-2-pyrollidone (NMP, 99.5%), potassium hydroxide (KOH, 85%) and lithium hydroxide (LiOH, 98%). All these chemical reagents were purchased from Merck (Johannesburg, South Africa) and used without further purification. Nickel foam was purchased from Material Technology International Corporation (MTI Corp., Richmond, CA, USA).

### 2.2. Synthetic Procedures

#### 2.2.1. Synthesis of Mn_3_O_4_ Electrode Material

**Figure 1:**
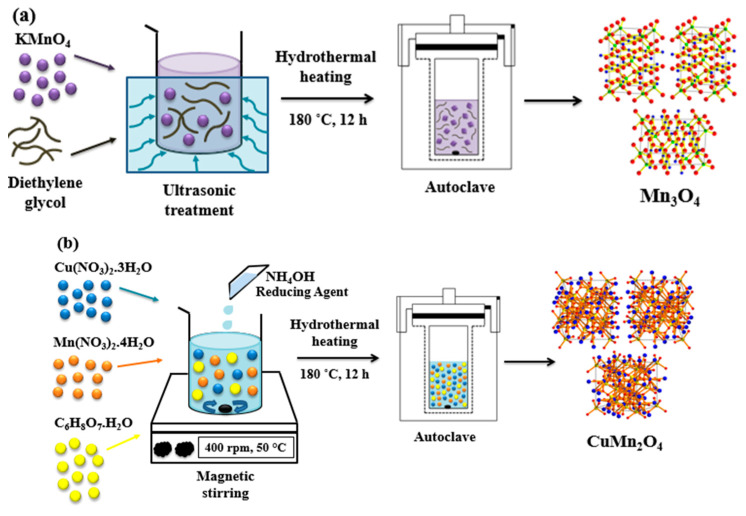

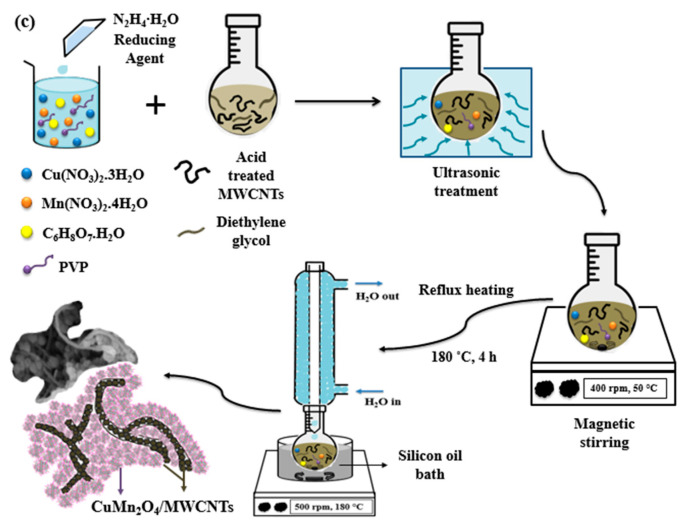
Experimental diagram for the synthetic procedures of Mn_3_O_4_ (**a**), CuMn_2_O_4_ (**b**) and CuMn_2_O_4_/MWCNTs (**c**) electrode materials.

The pristine electrode material was synthesized through a hydrothermal method. An amount of 1.50 g of KMnO_4_ was dispersed in 50 mL of ultra-pure water and subjected to ultrasonic treatment for 30 min followed with the addition of 10 mL of diethylene glycol. The mixture was transferred into a 100 mL Teflon-lined stainless-steel autoclave and heated at 180 °C for 12 h. After cooling to room temperature, the mixture was filtered by means of centrifugation and washed repeatedly with ethanol and distilled water. The solid product was dried overnight in a vacuum oven at 60 °C. The experimental diagram for the synthetic procedures of all the materials is displayed in Figure 1.

#### 2.2.2. Synthesis of CuMn_2_O_4_ Nanoparticles

1.55 g of Cu(NO_3_)_2_·3H_2_O was dissolved in 6 mL of dilute HNO_3_. To this solution was added 2.5 g of Mn(NO_3_)_2_·4H_2_O, 0.67 g of C_6_H_8_O_7_·H_2_O and 40 mL of ultra-pure water. The mixture was allowed to stir for 30 min followed by a drop wise addition of NH_4_OH which brought the solution pH to 8 prior to heating in an autoclave at 180 °C for 12 h. The solid was collected by centrifuging and washing with ethanol and distilled water. This product was vacuum-dried overnight at 60 °C and subsequently annealed in a muffle furnace at 400 °C for 4 h.

#### 2.2.3. Synthesis of CuMn_2_O_4_/MWCNTs

Firstly, MWCNTs were oxidized by adding 10 mg of raw MWCNTs to a concentrated acid- mixture of H_2_SO_4_ (98%) and HNO_3_ (65%) in a 3:1 ratio. The oxidation of the MWCNTs reduces their hydrophobicity and improves their wettability and solubility in polar media [30]. An amount of 5 mg of the acid-treated MWCNTs was dispersed in 60 mL of diethylene glycol for subsequent use. Thereafter, 1.55 g Cu(NO_3_)_2_·3H_2_O, 2.5 g Mn(NO_3_)_2_·4H_2_O, 0.67 g of C_6_H_8_O_7_·H_2_O and 0.4 g polyvinylpyrrolidone (PVP) were dissolved in 40 mL ultra-pure water and the solution pH adjusted by the dropwise addition of 10 mL N_2_H_4_·H_2_O to pH 8. To this solution was added the previously dispersed MWCNTs and the mixture subjected to ultrasonic treatment and magnetic stirring for 30 min followed by refluxing at 180 °C for 4 h. A black precipitate was collected after centrifuging and washing several times with deionised water and ethanol. The product was dried overnight at 60 °C in a vacuum oven and finally annealed in a muffle furnace at 500 °C for 5 h to obtain the CuMn_2_O_4_/MWCNTs. electrode material.

### 2.3. Material Characterization

The phases and crystal structures of the electrode materials were investigated by X-ray powder diffraction (XRD) using a focused D8 Advanced Diffractometer (Bruker AXS GmbH, Karlsruhe, Germany) equipped with a copper Kα radiation (λ = 1.54056 Å) source, scanning from 10° to 90° at an operating voltage of 40 kV and current of 40 mA. The crystallite size calculations were performed from the data extrapolation of the most intense Bragg diffraction peak. The surface morphology of electrode materials were explored via scanning electron microscopy (SEM) using a Zeiss Ultra Scanning Electron Microscope (Carl Zeiss AG, Oberkochen, Germany). Internal structural images were obtained via high-resolution transmission electron microscopy (HR-TEM) by a Tecnai G^2^F_2_O X-Twin MAT 200 kV Field Emission Transmission Electron Microscope from FEI (Eindhoven, Netherlands). Functional groups present in the electrode materials were examined using a Perkin Elmer Spectrum 100 Series Attenuated Total Reflectance Fourier Transform Infrared (ATR–FTIR) spectrometer (PerkinElmer Incorporated, Waltham, MA, USA). Bond vibrations and rotation information of the functional groups were obtained by employing Raman spectroscopy using a HORIBA Scientific XploRA PLUS Raman Microscope (HORIBA, Northampton, UK) equipped with a 532 nm laser source and 1 μm resolution. Small-angle X-ray scattering (SAXS) measurements of the pristine and modified electrode materials were conducted with an Anton Paar SAXSpace spectrometer (Anton Paar, Graz, Austria), equipped with a copper Kα radiation (λ = 1.54056 Å) source.

### 2.4. Electrochemical Measurements

Cyclic voltammetry (CV), galvanostatic charge-discharge (GCD), and electrochemical impedance spectroscopy (EIS) experiments were conducted in a three-electrode cell configuration, in both 3 M KOH and 3 M LiOH aqueous electrolytes, at room temperature on a BioLogic VMP-300 (BioLogic, Seyssinet-Pariset, France) electrochemical workstation. The three-electrode half-cell consisted of the working electrode (active material coated onto nickel foam substrate), reference electrode (Ag/AgCl in 3 M KCl), and the platinum wire counter electrode. The working electrodes were prepared by mixing the active electrode materials with carbon black (CB) conducting agent and polyvinylidene fluoride (PVDF) binder in a 70:20:10 weight percent ratio, followed by the addition of a few drops of anhydrous N-methyl-2-pyrrolidone (NMP) to form a homogeneous slurry. The slurry was coated onto the nickel foam and dried at 80 °C for 12 h. CV curves were obtained for the three-electrode cells within the voltage ranges of 0–0.7 V and 0–0.8 V, at specific applied scan rates of 5, 10, 20, 50, and 100 mV s^−1^. GCD experiments were conducted within the potential range of 0–0.6 V at current loadings of 0.5, 1, 2, 3, and 4 A g^−1^. The experimental data for EIS analysis was acquired within the frequency range of 10 mHz to 100 kHz with a sinusoidal perturbation amplitude of 5 mV at a 0.324 V applied DC potential.

## 3. Results and Discussion

### 3.1. Scanning Electron Microscopy (SEM)

**Figure 2:**
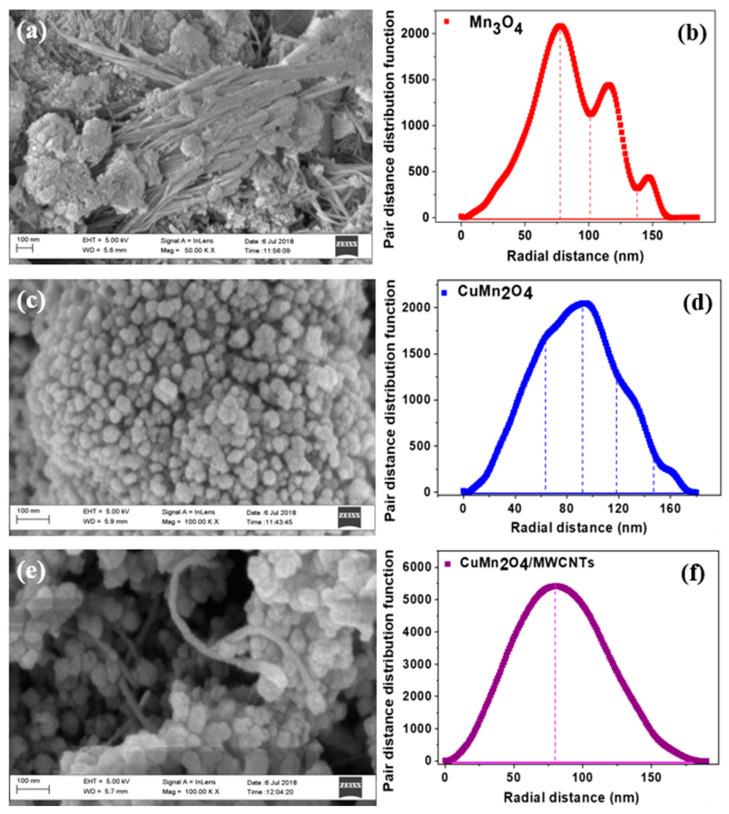
SEM image of Mn_3_O_4_ (**a**) viewed at a 100 nm scale, with the corresponding PDDF profile (**b**). SEM image of CuMn_2_O_4_ (**c**) at the 100 nm scale with its related PDDF profile (**d**). SEM image of CuMn_2_O_4_/MWCNTs (**e**) at the 100 nm scale with its complementary PDDF profile (**f**).

The surface morphologies of the Mn_3_O_4_, CuMn_2_O_4_ and CuMn_2_O_4_/MWCNTs electrode materials were investigated by SEM, as displayed in Figure 2. The Mn_3_O_4_ material shows aggregated particles with elongated and plate-like morphology as seen in Figure 2a, with average particle sizes ranging between 65–105 nm. The image also displays large secondary particles of approximately 145 nm originating from the aggregation of smaller primary particles. The particle distribution is in agreement with the SAXS profile shown in Figure 2b, representing the pair-distance distribution function (PDDF) of the Mn_3_O_4_. The PDDF profile of Mn_3_O_4_ illustrates small-angle scattering patterns showing average particle sizes between 60 and 90 nm. However, a decrease in the scattering fraction number (*P*) is observed within the particle size range of 110–160 nm, indicating aggregation of the bigger Mn_3_O_4_ nanoparticles. The CuMn_2_O_4_ morphology displayed in Figure 2c shows clusters of geometric nanoparticles with calculated average particle sizes ranging between 75–120 nm. The nanopores in the material allow for efficient diffusion of electrolyte ions throughout the material surface. The PDDF profile of the CuMn_2_O_4_ material, shown in Figure 2d, illustrates spherically shaped nanoparticles having particle sizes ranging between 70–110 nm and with less particle aggregation due to increased stability of CuMn_2_O_4_ nanostructures confirmed from the XRD analysis. The morphology of CuMn_2_O_4_/MWCNTs, presented in Figure 2e, shows the clustered CuMn_2_O_4_ geometric nanoparticles intertwined with the MWCNTs which serve as alternative diffusion pathways for electrolyte ions to traverse the material for faster electrochemical kinetics and rate capability [30]. The CuMn_2_O_4_/MWCNTs has a broad and symmetrical PDDF profile as shown in Figure 2f indicating uniform distribution of particles with average sizes between 50–108 nm.

### 3.2. High-Resolution Transmission Electron Microscopy (HR-TEM)

**Figure 3:**
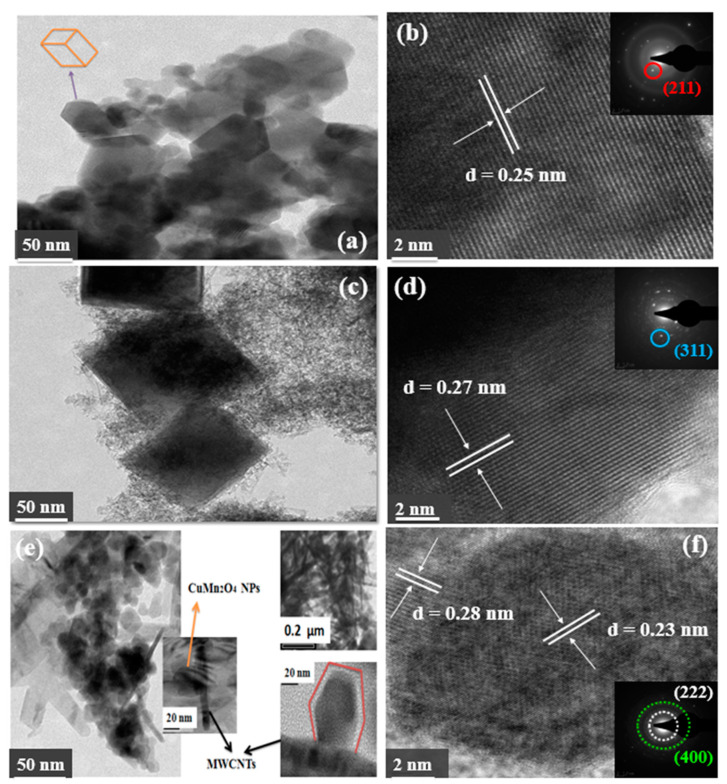
HR-TEM images of Mn_3_O_4_ viewed at 50 nm scale (**a**) and 2 nm scale (**b**). HR-TEM images of CuMn_2_O_4_ at the 50 nm scale (**c**) and 2 nm scale (**d**). HR-TEM images of CuMn_2_O_4_/MWCNTs at the 50 nm scale (**e**) and 2 nm scale (**f**).

HR-TEM images of the Mn_3_O_4_ material in Figure 3a,b show rectangular-shaped agglomerated particles with atomic planes represented by lattice fringes. The selected area diffraction (SAED) pattern displayed as an inset in Figure 3b, illustrates a particular sequence of diffracted spots characteristic to Mn_3_O_4_ structures [31]. The HR-TEM image viewed at a 2 nm scale, in Figure 3b, shows lattice fringes representing atomic lattice planes with the indexed planes having an inter-planar spacing distance of 0.25 nm. This *d*-spacing distance corresponds to the *d*-space distance of the (211) indexed Bragg diffraction peak from the Mn_3_O_4_ XRD pattern. The CuMn_2_O_4_ nanoparticles displayed in Figure 3c, show rhombic nanostructures with particle sizes ranging between 95–140 nm. The SAED pattern of the CuMn_2_O_4_ material, inset in Figure 3d, illustrates high crystallinity resulting from the brighter and closely oriented diffracted spots. The HR-TEM image of the CuMn_2_O_4_/MWCNTs material, displayed in Figure 3e, shows the rhombic CuMn_2_O_4_ nanoparticles anchored to the MWCNTs surface. Image processing analysis revealed the CuMn_2_O_4_/MWCNTs material having average particle sizes ranging between 50–108 nm. In the SAED pattern of CuMn_2_O_4_/MWCNTs, the diffracted spots formed symmetric rings each arising from their respective atomic planes. This observation indicates that the CuMn_2_O_4_/MWCNTs material possesses a polynanocrystalline phase [32]. The indexed atomic lattice planes of (222) and (400) in the SAED pattern of CuMn_2_O_4_/MWCNTs corresponds to the CuMn_2_O_4_ phases present within the nanostructure as confirmed by the two highly intense Bragg diffraction peaks indexed in the CuMn_2_O_4_/MWCNTs XRD pattern. The EDS spectra of all the materials are shown in Figure S1. All the elements in Mn_3_O_4_ are present in the spectrum. However, the K and Si signals are due to residues and contaminants from the starting materials. The Cu in the EDS spectrum of Mn_3_O_4_ nanoparticles emanated from the Cu grid, and the C signal observed came from the carbon coated onto the sample to improve contrast during analysis. The EDS spectrum of CuMn_2_O_4_ confirmed the presence of the constituent elements with the observed Ni signal originating from the Ni grid used during analysis. The EDS spectrum of the CuMn_2_O_4_/MWCNTs showed the presence of all the constituent elements.

### 3.3. Structural Characterisation

**Figure 4:**
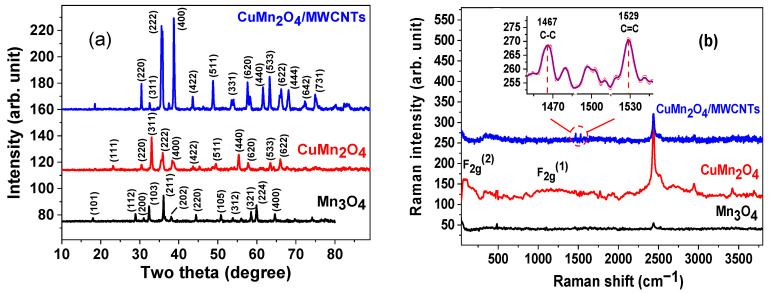

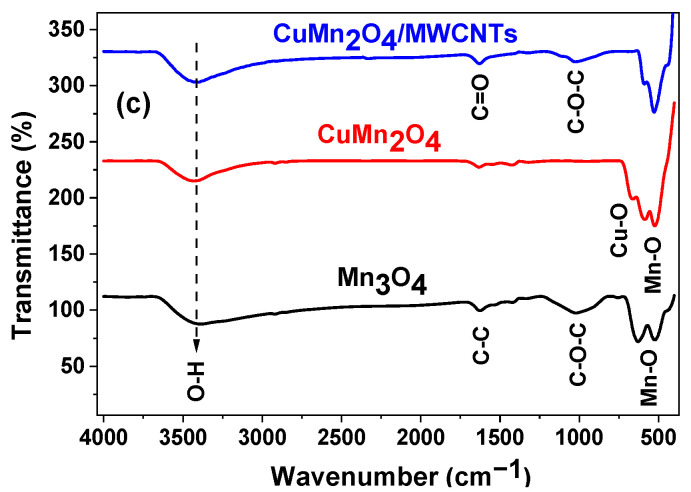
XRD patterns (**a**), Raman spectra (**b**) and FTIR spectra (**c**) of Mn_3_O_4_, CuMn_2_O_4_ and CuMn_2_O_4_/MWCNTs electrode materials.

The XRD patterns of the Mn_3_O_4_, CuMn_2_O_4_, and CuMn_2_O_4_/MWCNTs electrode materials, shown in Figure 4a, display sharp and intense Bragg diffraction peaks thus suggesting good crystallization of the synthesized electrode materials. The major diffraction peaks observed in the XRD pattern of Mn_3_O_4_ located at diffraction angles of 28.96°, 32.36°, 36.12° and 44.45° are indexed with miller indices of (112), (103), (211) and (220), respectively. The phase is identified as Mn_3_O_4_ from its Joint Committee on Powder Diffraction Standards (JCPDS) data file number 24–0734 (Hausmannite, syn), with a body-centred tetragonal (BCT) crystal structure and a 141/amd (141) space group. The XRD pattern of the CuMn_2_O_4_ illustrates broader diffraction peaks with higher intensities compared to the XRD pattern of Mn_3_O_4_. The high intensity diffraction peaks observed in the XRD pattern of CuMn_2_O_4_ located at 30.40°, 35.86°, 38.46° and 43.67° diffraction angles are indexed to the (220), (311), (222) and (400) planes, respectively. The XRD pattern identifies a face-centered cubic (FCC) crystal structure and Fd3m (227) space group of CuMn_2_O_4_ nanoparticles, which matches well with its JCPDS #: 84–0543 data file [33]. The XRD pattern of CuMn_2_O_4_/MWCNTs illustrates the highest intensity Bragg diffraction peaks at 2θ angles of 30.40°, 38.72°, 43.53° and 53.81° assigned with their respective (220), (222), (400) and (422) lattice planes. These atomic lattice planes are indexed according to the existence of a uniform CuMn_2_O_4_/MWCNTs phase with an Fd3m (227) space group characteristic of FCC crystal systems [33,34]. It is observed that the diffraction peaks of CuMn_2_O_4_/MWCNTs occur at 2θ positions analogous to that of the spinel CuMn_2_O_4_, implying they match well with one another. The sharper and narrower diffraction peaks of the composite CuMn_2_O_4_/MWCNTs indicate better crystallinity and electrochemical conductivity of the material as reflected in its lower charge transfer resistance compared to the Mn_3_O_4_ and CuMn_2_O_4_ in the EIS data. The crystallite size calculations were based on the Scherrer’s equation below [35]:

(1) $$D_{\left(h,k,l\right)}=\frac{K\lambda }{\beta \cos\theta }$$

where *D* is the mean crystallite size (nm), (*h, k*, *l*) are the miller indices indexed to the Bragg diffraction peak, *K* is the Scherrer’s constant (0.89), λ is the wavelength of the X-ray beam (0.154 nm), β is the full width at half maximum (FWHM) of the Bragg diffraction peak and θ is the Bragg’s angle.

Table 1 shows the values of *D*, inter-planar spacing distance (*d*) and lattice parameters (*a*) and (*c*) of the three electrode materials with the CuMn_2_O_4_/MWCNTs exhibiting the smallest crystallite size of 51.26 nm. This decreased size as the SAXS and TEM data also show implies a larger surface area and hence more available sites for electrolyte interaction thus leading to higher electrochemical performance as shown in the CV and EIS data. The CuMn_2_O_4_ has a crystallite size of 77.94 nm, which is slightly larger than that of Mn_3_O_4_ (74.04 nm). This can be attributed to the CuMn_2_O_4_ FCC crystal structure containing eight tetrahedral and four octahedral interstitial sites, at which the Cu^2+^ and Mn^3+^ transition metal ions are situated [20].

The Raman spectra of the Mn_3_O_4_, CuMn_2_O_4_ and CuMn_2_O_4_/MWCNTs were acquired for molecular bond stretching and vibration analyses. The spectrum of the Mn_3_O_4_ material shown in Figure 4b, illustrates a low intensity Raman peak at approximately 485 cm^−1^, characteristic to symmetric F_2g_^(2)^ stretching vibration modes of Mn–O species within the octahedral MnO_6_ sub-lattices of the Mn_3_O_4_ crystal structure [36]. The CuMn_2_O_4_ displays a more intense Raman-active bands than that of Mn_3_O_4_. The intensity of the broad Raman shoulder band (F_2g_^(1)^) along the shift range of 900–1500 cm^−1^ in the CuMn_2_O_4_ spectrum is closely related to the oxidation/valence state of Mn^3+^ atoms within the CuMn_2_O_4_ crystal structure [37]. This increase in the intensities of the CuMn_2_O_4_ active bands compared to Mn_3_O_4_ is attributable to the higher oxidation/valence state of Mn^3+^ ions within the CuMn_2_O_4_ crystal lattice. The Raman spectrum of the CuMn_2_O_4_/MWCNTs material illustrates two partially distinctive peaks, enlarged on the inset graph, positioned at 1467 cm^−1^ and 1529 cm^−1^, ascribed to the *D* and *G* bands, respectively. The *D* band is attributed to the breathing mode of *k*-point phonons of A_1g_ symmetry from the carbon aromatic rings, whereas the *G* band is assigned to the E_2g_ phonons from the stretching vibrations of sp^2^ hybridized carbon atoms in the MWCNTs [38,39]. The incorporation of CuMn_2_O_4_ nanoparticles to the MWCNTs network can be confirmed by observing the additional Raman E_g_ band at 362 cm^−1^. The high-intensity Raman peak occurring within the shift range of 2430–2435 cm^−1^ represents the 2*D* band, which is assigned to an overtone mode of longitudinal optical phonon from the dispersion and frequency of 2*k*-point phonons [40].

The FT-IR spectra of the Mn_3_O_4_, CuMn_2_O_4_ and CuMn_2_O_4_/MWCNTs are presented in Figure 4c. The broad absorption band, appearing consistently in each material’s spectrum, within the high frequency range of 3000–3660 cm^−1^ is ascribed to the bond stretching vibrational modes of hydroxyl (O–H) groups in water [41]. The intense absorption bands located at lower frequencies of 520 cm^−1^ and 623 cm^−1^ correspond to the vibrations in Mn–O bond within the Mn_3_O_4_ structure. The absorption bands located at wavenumbers 1123 cm^−1^ and 1720 cm^−1^ are characteristic to the C–O and C–C stretching vibrations, originating from diethylene glycol residues. In the spectrum of CuMn_2_O_4_, the two bands representing Mn–O bond vibrations appeared less intense due to vibrations from the neighbouring Cu–O bond, as illustrated by the faint absorption band at 660 cm^−1^. The FT-IR spectrum of the CuMn_2_O_4_/MWCNTs material shows the absorption bands corresponding to Mn–O and Cu–O functional groups, however, at weaker intensities due to C = O and C–O bond vibrations, at 1630 cm^−1^ and 1020 cm^−1^, derived from the carbon backbone of the MWCNTs network [14].

### 3.4. Electrochemical Characterization

#### 3.4.1. Cyclic Voltammetry (CV)

**Figure 5:**
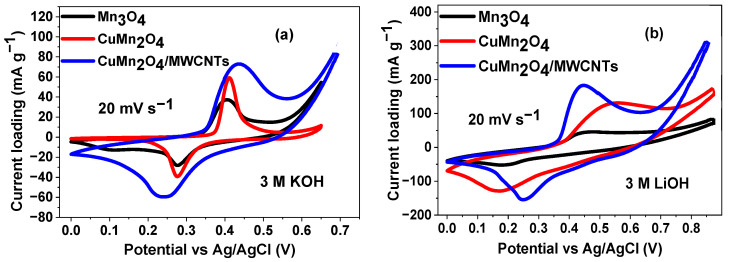

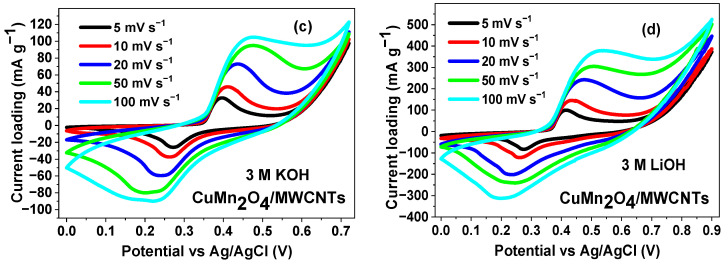
CV curves of individual materials scanned at 20 mV s^−1^ in 3 M KOH (**a**) and 3 M LiOH (**b**) aqueous electrolytes; CV curves of CuMn_2_O_4_/MWCNTs electrode material in 3 M KOH (**c**) and 3 M LiOH (**d**) aqueous electrolytes at different scan rates.

Figure 5 shows the cyclic voltammograms of Mn_3_O_4_, CuMn_2_O_4_ and CuMn_2_O_4_/MWCNTs electrode materials. Each CV is characterised by a pair of redox peaks arising from the Mn^2+^/Mn^3+^ and Mn^3+^/Mn^4+^ redox couples. The redox reaction mechanisms of the electrode materials are presented as follows [42,43].

(2) $${\mathrm{Mn}}_{3}O_{4}+{\mathrm{OH}}^{-}+H_{2}O⇌3\mathrm{MnOOH}+e^{-}$$

(3) $${\mathrm{CuMn}}_{2}O_{4}+{\mathrm{OH}}^{-}+H_{2}O⇌2\mathrm{MnOOH}+\mathrm{CuOOH}+e^{-}$$

(4) $$\mathrm{CuOOH}+{\mathrm{OH}}^{-}⇌{\mathrm{CuO}}_{2}+H_{2}O+e^{-}$$

The CuMn_2_O_4_/MWCNTs showed better electrochemical performance in both electrolytes than the other electrode materials as observed in Figure 5a,b. This is attributed to the three-dimensional support structure of the MWCNTs that stabilized the electroactive sites and promoted more electrolyte ion diffusion. Figure 5c,d describe the effect of potential scan rates on the CV of the CuMn_2_O_4_/MWCNTs in each electrolyte. Scanning from 5–100 mV s^−1^, the current was observed to increase as the scan rate increases, with the anodic peaks shifting to more positive potentials and the cathodic peaks to more negative potentials; a phenomenon that shows that the electrochemical process on the electrode/electrolyte interface is quasi-reversible with the CuMn_2_O_4_/MWCNTs electrode exhibiting a higher current response.

It is known that charge storage in pseudocapacitors involves redox processes with capacitive behaviour [44], and since a capacitive current follows a linear dependence on scan rate, Figure S2a,b indicate that the pseudocapacitance in the CuMn_2_O_4_/MWCNTs electrode material is a surface-controlled electrochemical process governed by the equation [45].

(5) i=dQdt=CdEdt=Cν

where *Q* is the voltammetric charge, *C* is the capacitance and dEdt is the scan rate, ν. This pseudocapacitive behaviour is corroborated by the results from the log-log plot of current against scan rate as shown in Figure S2c,d, obtained according to the power law relationship [46].

(6) $$i=a\nu^{b}$$

where *a* and *b* are adjustable parameters. The value of *b* provides important information about the charge-storage kinetics. When *b* is 1, the charge storage mechanism is highly capacitive and when *b* is 0.5, the process is diffusion-controlled. The CuMn_2_O_4_/MWCNTs has a *b* value of 0.86 and shows a more pseudocapacitive charge-storage mechanism in LiOH than in KOH where the *b* value is 0.58 suggesting a more diffusion-controlled kinetics. The charge storage capability of the different electrodes was evaluated by determining the specific capacitance from the integrated area under the CV curves in Figure 5c,d. Values were obtained according to the equation [47]:

(7) $$Csp=\frac{\int_{V_{a}}^{V_{c}}I(V)dV}{\nu m(V_{c}-V_{a})}$$

where *C*_sp_ is the specific capacitance (F g^−1^) of the electrode materials, *V*_a_ and *V*_c_ are the two potential limits (V) of the integrated area under the CV curves, *I* is the corresponding current (A) obtained from the current density, *m* is the average mass loading (g) of electroactive materials and ν is the potential scan rate (V s^−1^).

Figure S3a shows the plot of specific capacitance against scan rates for the CuMn_2_O_4_/MWCNTs electrode material in KOH and LiOH. The specific capacitance values obtained at the lowest applied scan rate of 5 mV s^−1^ are 659.71 F g^−1^ for KOH and 1267.43 F g^−1^ for LiOH. This also confirms the earlier observations made that electrochemical performance of the electrode materials in LiOH is better than in KOH. In their work with MnO_2_ in different aqueous hydroxides, Misnon et al. [48] reported the highest specific capacitance value in LiOH than in KOH and NaOH and attributed it to the smaller ionic radius of Li^+^ that allowed easier intercalation-de-intercalation reaction to occur. The specific capacitance is higher at lower scan rates and decreased as the scan rate progresses. This is due to the fact that at lower scan rates, more electroactive sites are accessible by the electrolyte ion whereas at higher scan rates, the penetration distance of the electrolyte ion into the material decreases and the ions are only limited to the surface. Figure S3a also shows that the CuMn_2_O_4_/MWCNTs electrode analysed in LiOH retained about 73% of its initial specific capacitance after increased scan rates from 5–100 mV s^−1^, whereas analysis of the nanocomposite cathode in KOH aqueous electrolyte has a capacitance retention of 43% thus indicating enhanced ionic interactions of Li^+^ which resulted in higher rate capabilities. Figure S3b illustrates the comparative CVs of the CuMn_2_O_4_/MWCNTs electrode before and after 6000 cycles in 3 M LiOH. The specific capacitance recorded at 100 mV s^−1^ gave a value of 643.12 F g^−1^ after 6000 cycles, which is about 70% of its initial capacitance before cycling. This result indicates a relatively good electrochemical stability of the CuMn_2_O_4_/MWCNTs electrode. Figure S3c shows the CVs of the bare MWCNTs electrodes in both electrolytes. The CVs are quasi-rectangular in shape, with no discernible electrochemistry, indicating the supercapacitor behaviour. The CVs of Mn_3_O_4_ at various scan rates are shown in Figure S4.

#### 3.4.2. Electrochemical Impedance Spectroscopy (EIS)

**Figure 6:**
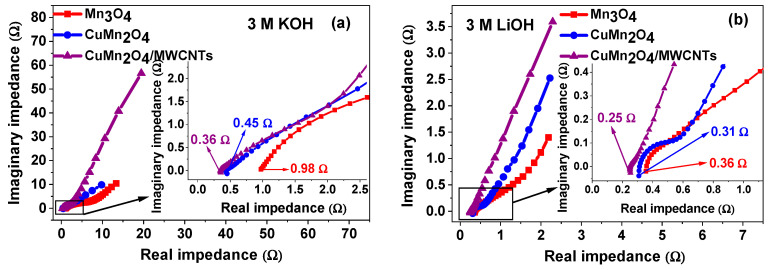

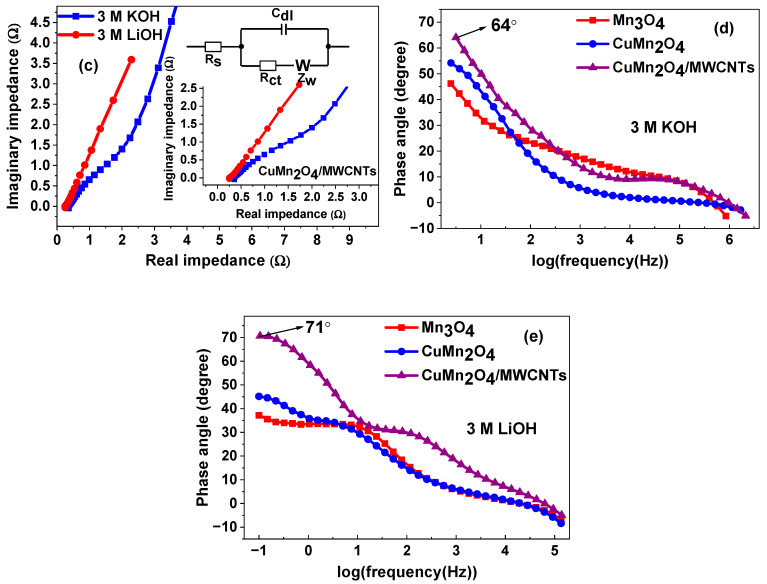
Nyquist plots of all analysed electrode materials in 3 M KOH (**a**) and 3 M LiOH (**b**) aqueous electrolytes; comparative Nyquist plot of CuMn_2_O_4_/MWCNTs in both 3 M KOH and 3 M LiOH aqueous electrolytes (**c**); corresponding Bode phase impedance plot of all electrode materials in 3 M KOH (**d**) and 3 M LiOH (**e**) aqueous electrolytes.

Electrochemical impedance spectroscopy was used to shed more light on the charge transfer behaviour and the capacitive nature of the electrode materials. In Figure 6 the Nyquist and Bode plots of the pristine Mn_3_O_4_, spinel CuMn_2_O_4_, and CuMn_2_O_4_/MWCNTs electrode materials in both electrolytes are shown. In the high-frequency region of a typical Nyquist plot, the intercept of the curve at the x–axis refers to the bulk solution resistance of the electrode material at the electrode/electrolyte interface [43,49]. This bulk solution resistance (*R*_s_) involves intrinsic resistances experienced by the electroactive material which include ionic resistances from electrolyte ions at the electrode/electrolyte interface, as well as contact surface resistances occurring between the electroactive species and Ni-foam (substrate) current collector. The diameter of the semi-circle of impedance is equivalent to the charge-transfer resistance (*R*_ct_) in the high frequency region, with the slanted line occurring in the low frequency region representing the Warburg impedance (*Z*_w_), which mathematically expresses the diffusion of electrolyte ions according to an equivalent circuit model. The *R*_ct_ value signifies the resistance of electron transfer kinetics occurring at the electrode/electrolyte interface during charging/discharging processes. The double layer capacitance (*C*_dl_) models the effect of charge accumulation in electrolyte ions at the electrode material’s surface [50,51]. The values of the kinetic parameters from EIS for all the analysed electrode materials in both electrolytes are summarised in Table 2 and Table 3.

The Nyquist plots of Mn_3_O_4_, CuMn_2_O_4_ and CuMn_2_O_4_/MWCNTs in KOH electrolyte are shown in Figure 6a. The Mn_3_O_4_ is characterised by a larger semi-circle at high frequency with the highest *R*_ct_ value and an indistinct Warburg diffusion line at the low frequency region, whereas the CuMn_2_O_4_ and CuMn_2_O_4_/MWCNTs have diminished semi-circles at higher frequency which is confirmed by their lower *R*_ct_ values indicating better electron transfer kinetics at the electrode/electrolyte interface. Both also have the Warburg diffusion line at low frequency which is, however, more prominent in CuMn_2_O_4_/MWCNTs than in the CuMn_2_O_4_ electrode. The Warburg line that is near-parallel to the imaginary impedance axis suggests a more pseudocapacitive behaviour in the CuMn_2_O_4_/MWCNTs. The more vertical the curve, the more a supercapacitor (or pseudocapacitor) behaves like an ideal capacitor [52,53,54,55] and lower Z_w_ values suggest that the kinetics of charge storage is capacitive [56].

The inset in the figure shows an expanded portion of the high frequency region indicating the *R*_s_ values of the electrodes. The behaviour of the electrode materials in LiOH electrolyte, shown in Figure 6b, follow an almost similar trend as observed in KOH. Each electrode is characterised by a semi-circle in the high frequency region and an inclined Warburg line at low frequency. The *R*_ct_ and *R*_s_ values decrease from Mn_3_O_4,_ CuMn_2_O_4_ to CuMn_2_O_4_/MWCNTs as shown in Table 2. The straight line in the low frequency region is near-parallel to the imaginary impedance axis, especially for the CuMn_2_O_4_/MWCNTs electrode which again, signifies the characteristics of a pseudocapacitor. The enlarged portion of the high frequency region is shown in the inset. Figure 6c compares the Nyquist plots of the CuMn_2_O_4_/MWCNTs in both electrolytes. Each plot is marked by a semi-circle at high frequency and a Warburg impedance at low frequency. The figure shows that the CuMn_2_O_4_/MWCNTs electrode material exhibited a more vertical Warburg line in LiOH than in KOH aqueous electrolyte, thus indicating a more pseudocapacitive behaviour in the former.

The Bode phase angle profiles of the materials analysed in KOH are shown in Figure 6d. The CuMn_2_O_4_/MWCNTs has the highest phase angle followed by the CuMn_2_O_4_ and Mn_3_O_4_ as presented in Table 2. It is well known that an ideal capacitor has a phase angle of about 90° [53,57]. The nanocomposite electrode with the highest phase angle that tends towards that of an ideal capacitor can be said to exhibit a more pseudocapacitive character than the others. The increased electrochemical performance is attributed to the synergy between the spinel CuMn_2_O_4_ and MWCNTs which increased the surface conductivity, fast ion transport and sufficient number of active sites, leading to higher adsorption of ions and better performance [21,54,56]. A similar observation was made in Figure 6e for the analysis of the materials in LiOH.

**Figure 7:**
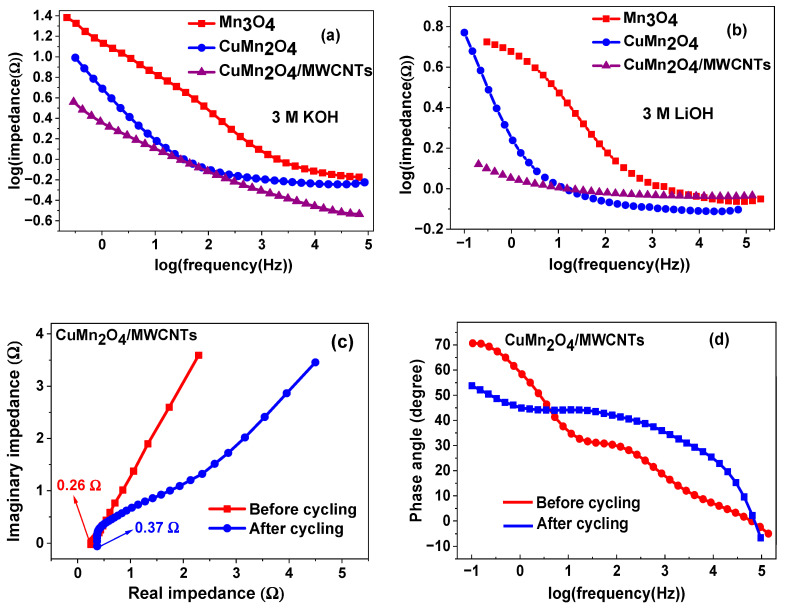
Graphs of log impedance against log frequency for all analysed electrode materials in 3 M KOH (**a**) and 3 M LiOH (**b**) aqueous electrolytes; Nyquist (**c**) and Bode (**d**) plots of CuMn_2_O_4_/MWCNTs electrode material before and after 6000 cycles in 3 M LiOH aqueous electrolyte.

The log impedance against log frequency diagrams in Figure 7a,b illustrated a lower impedance in the electrodes for each electrolyte group and with the electrodes in LiOH having the least impedance overall. This result is further confirmed by the faster angular frequency response-time (τ) exhibited by the CuMn_2_O_4_/MWCNTs electrode material thus indicating more efficient ion transport routes enabled by the MWCNTs throughout the CuMn_2_O_4_/MWCNTs matrix. The equivalent circuit model, displayed as an inset in Figure 6c, was used to perform EIS data fitting using an EC-Lab EIS Z-fit software. The Nyquist and Bode impedance diagrams in Figure 7c,d, showed a 32% increase and a 27% decrease in resistance and conductivity of the CuMn_2_O_4_/MWCNTs electrode after 6000 cycles in 3 M LiOH aqueous electrolyte. The Nyquist and Bode impedance plots of the bare MWCNTs electrode, shown in Figure S6a,b, illustrates a lower charge transfer resistance and higher phase angle in LiOH compared to KOH aqueous electrolyte. The values of the kinetic parameters from EIS for the bare MWCNTs electrode in both KOH and LiOH electrolytes are summarised in Table S1. Comparative Bode plots between the CuMn_2_O_4_/MWCNTs electrode in KOH and LiOH is presented in Figure S6c with the CuMn_2_O_4_/MWCNTs in LiOH being more pseudocapacitive because of the higher phase angle value as shown in Table 2 and Table 3.

#### 3.4.3. Galvanostatic Charge/Discharge (GCD)

**Figure 8:**
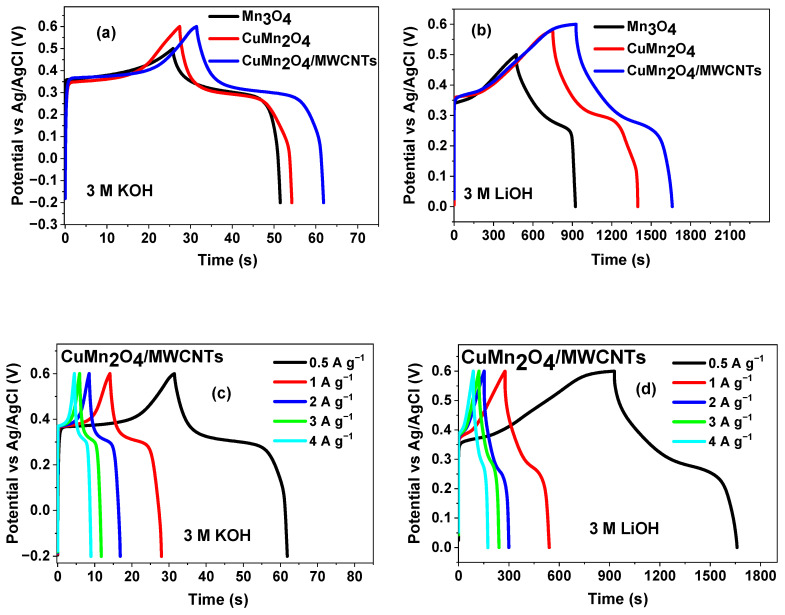
GCD curves of all electrode materials obtained at 0.5 A g^−1^ in 3 M KOH (**a**) and 3 M LiOH (**b**) aqueous electrolytes; GCD curves of CuMn_2_O_4_/MWCNTs electrode material in 3 M KOH (**c**) and 3 M LiOH (**d**) aqueous electrolytes.

Figure 8 compares the galvanostatic charge/discharge profiles of the electrode materials. Figure 8a–d show typical charge/discharge curves of the materials at different current densities and in different electrolytes. All the three electrodes exhibited better electrochemical performance in LiOH as also observed in the CV results. The equation below was used to calculate the specific capacitance at various current loadings [58]. Table 4 shows the comparison of supercapacitance values of electrode materials evaluated in LiOH and KOH aqueous electrolytes.

(8) $$C_{\mathrm{sp}}=\frac{I\Delta t}{\Delta Vm}$$

where *C*_sp_ is the specific capacitance (F g^−1^), *I* is the discharge current (A), Δ*t* is the discharge time (s), Δ*V* is the cell operating potential window (V) and *m* is the average mass of active electrode materials coated onto the Ni-foam current collector.

**Figure 9:**
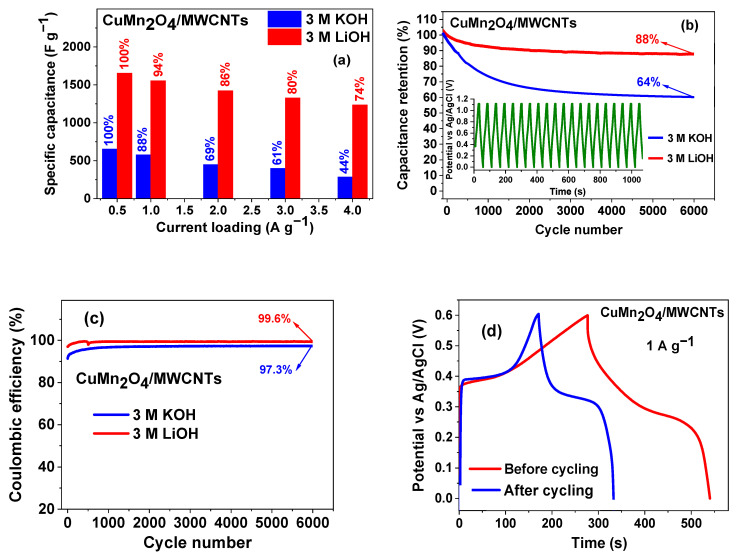
Histogram of the specific capacitance of CuMn_2_O_4_/MWCNTs electrode material in 3 M KOH and 3 M LiOH aqueous electrolytes (**a**); cycling performance of CuMn_2_O_4_/MWCNTs electrode material in 3 M KOH and 3 M LiOH aqueous electrolytes after 6000 cycles at 1 A g^−1^ current load (**b**); Coulombic efficiency of CuMn_2_O_4_/MWCNTs electrode material in 3 M KOH and 3 M LiOH aqueous electrolytes over 6000 cycles obtained at 1 A g^−1^ current loading (**c**); GCD curves of CuMn_2_O_4_/MWCNTs electrode material before and after 6000 cycles at 1 A g^−1^ in 3 M LiOH aqueous electrolyte (**d**).

The histogram shown in Figure 9a illustrates the values of the specific capacitances of the CuMn_2_O_4_/MWCNTs electrode material at various current loads ranging between 0.5–4 A g^−1^, for both electrolytes. At all current loads, the material analysed in LiOH gave the highest specific capacitance. The histogram shows the CuMn_2_O_4_/MWCNTs electrode retaining 74% of its initial specific capacitance when analysed in LiOH while the CuMn_2_O_4_/MWCNTs electrode examined in KOH sustained 44% of its initial specific capacitance. The GCD curves of Mn_3_O_4_ at various current loadings shown in Figure S5 illustrates the electrode material exhibiting a longer discharge time and higher specific capacitance in LiOH (450.86 s and 853.15 F g^−1^) compared to KOH (79.69 s and 56.58 F g^−1^) aqueous electrolytes. GCD curves of the bare MWCNTs, shown in Figure S6, indicates a higher specific capacitance performance in LiOH (50.37 F g^−1^) as compared to KOH (38.66 F g^−1^) at 2 A g^−1^.

The cycling stability of the CuMn_2_O_4_/MWCNTs was tested in both electrolytes as shown in Figure 9b. For the CuMn_2_O_4_/MWCNTs in LiOH electrolyte, a slight capacitance loss was noticed at the beginning of the cycling process but later stabilized around the 200th cycle and remained constant thereafter. The initial capacitance loss was more obvious for CuMn_2_O_4_/MWCNTs in KOH up to the 1000th cycle and became stable afterwards. After 6000 cycles, the CuMn_2_O_4_/MWCNTs electrode showed a specific capacitance retention of 88% and 64% in LiOH and KOH electrolytes, respectively, at 1 A g^−1^ current load. Overall, the CuMn_2_O_4_/MWCNTs electrode’s cycle stability and increased higher specific capacitance resulted in a greater charge storage capability and longer cycle life [67,68].

The Coulombic efficiency per cycle number of the CuMn_2_O_4_/MWCNTs electrode material in both 3 M KOH and 3 M LiOH aqueous electrolytes is shown in Figure 9c. It is observed from the graph that the electrode material in KOH electrolyte exhibited a 91.5% Coulombic efficiency for the first cycle, up to 97.3% at the 6000th cycle. On the other hand, the composite electrode material in LiOH electrolyte exhibited a 96.9% Coulombic efficiency at the first cycle and up to 99.6% at the 6000th cycle. This result also indicates that the cycling stability of the CuMn_2_O_4_/MWCNTs electrode material is better in the LiOH electrolyte than in the KOH electrolyte. Although the size of the hydrated cation K^+^ is smaller than that of Li^+^ (K^+^: 3.31 Å; Li^+^: 3.82 Å) which confers a higher mobility and conductivity in KOH electrolyte than in LiOH electrolyte, the higher capacitance values and superior performance in the LiOH electrolyte than in the KOH electrolyte is attributed to an easy intercalation/de-intercalation of Li^+^ ions into the electrode due to the smaller radius of Li^+^ (crystal size: 0.60 Å) than K^+^ (crystal size: 1.33 Å) which increased the double layer as well as pseudocapacitance [48]. Since intercalation and de-intercalation of alkaline electrolyte ions is generally involved for pseudocapacitive materials, the bare (unsolvated) ionic size is considered to have a pronounced effect on the pseudocapacitive behaviour [69]. Yuan and co-worker [70] reported a higher specific capacitance and cyclic stability in LiOH electrolyte than in KOH electrolyte and ascribed it to the Li^+^ insertion/extraction in MnO_2_ solid which is different with that electrode in KOH. This phenomenon was also corroborated in the work of Manickam et al. [71]. In a related study, Inamdar et al. [72] prepared NiO and recorded a specific capacitance that was almost two times higher in NaOH (Na^+^ crystal radius: 0.95 Å) than in KOH electrolyte. The authors attributed this to a higher intercalation rate of Na^+^ ions in the NIO electrode. It is well known that electrochemical stability is strongly related to cycle life [29], hence, the higher cyclability witnessed in LiOH indicates that the electrode has better stability and longer cycle life after repeated charge/discharge in this electrolyte compared to the KOH electrolyte. The GCD curves of CuMn_2_O_4_/MWCNTs before and after 6000 cycles in LiOH electrolyte is shown in Figure 9d and gave a discharge time and specific capacitance value of 130.4 s and 409.25 F g^−1^, respectively.

## 4. Conclusions

In this study, Mn_3_O_4_, CuMn_2_O_4_ and CuMn_2_O_4_/MWCNTs electrode materials were prepared. Results from XRD revealed the Mn_3_O_4_ material with a body-centred tetragonal (BCT) crystal structure, whereas both the CuMn_2_O_4_ and CuMn_2_O_4_/MWCNTs materials belong to the face-centred cubic (FCC) crystal systems. The crystal lattice structures of the Mn_3_O_4_ and CuMn_2_O_4_ materials are supported by their respective HR-TEM images, portraying two-dimensional rectangular-shaped Mn_3_O_4_ nanoparticles and slightly distorted rhombic CuMn_2_O_4_ nanostructures. Studies on the electrochemical properties of the electrode materials were conducted in both 3 M KOH and 3 M LiOH electrolytes. CV and EIS measurements illustrated a superior electrochemical performance in the nanocomposite electrode material compared to the CuMn_2_O_4_ and Mn_3_O_4_ electrode materials. The nanocomposite gave smaller charge transfer resistances in both electrolytes than the Mn_3_O_4_ and CuMn_2_O_4_ electrode materials and better electrochemical performance than the Mn_3_O_4_ and CuMn_2_O_4_ electrode materials. This is ascribed to the synergy between the spinel CuMn_2_O_4_ and MWCNTs which increased the surface conductivity, fast ion transport and sufficient number of active sites, leading to higher adsorption of ions and better performance. The results also revealed a better electrochemical response for all the electrode materials in the LiOH electrolyte than in the KOH electrolyte, attributed to faster intercalation/de-intercalation of Li^+^ ions into the materials. A specific capacitance of 1652.91 F g^−1^ at 0.5 A g^−1^ specific current was recorded for the CuMn_2_O_4_/MWCNTs electrode in the LiOH electrolyte while in the KOH electrolyte, the specific capacitance calculated was 653.41 F g^−1^ at 0.5 A g^−1^. The electrode also showed a higher cycling stability in the LiOH electrolyte with a 99.6% Coulombic efficiency, and looks more promising as an electrode material for pseudocapacitor applications in this electrolyte than in the KOH electrolyte, where it exhibited a lower charge storage behaviour.

## Figures and Tables

**Table 1:** Summary of crystallographic parameters calculated from the XRD of the Mn_3_O_4_ CuMn_2_O_4_, and CuMn_2_O_4_/MWCNTs electrode materials.

Electrode Material	*D* (nm)	*d* (nm)	*a* (nm)	*c* (nm)
Mn_3_O_4_	74.04	0.20	0.58	0.95
CuMn_2_O_4_	77.94	0.27	0.94	
CuMn_2_O_4_/MWCNTs	51.26	0.23	0.77	

**Table 2:** EIS fitted data of the Mn_3_O_4_, CuMn_2_O_4_ and CuMn_2_O_4_/MWCNTs electrode materials in 3 M KOH aqueous electrolyte.

Electrode Material	*R*_s_ (Ω)	*C*_dl_ (F)	*R*_ct_ (Ω)	*Z*_w_ (Ω s^−1/2^)	*τ* (s rad^−1^)	(°)
Mn_3_O_4_	0.98	2.58 × 10^−3^	1.29	4.98	4.62 × 10^−3^	46
CuMn_2_O_4_	0.45	4.64 × 10^−3^	0.83	2.86	3.83 × 10^−3^	55
CuMn_2_O_4_/MWCNTs	0.36	7.93 × 10^−3^	0.63	0.86	3.34 × 10^−3^	64

**Table 3:** EIS fitted data of the Mn_3_O_4_, CuMn_2_O_4_ and CuMn_2_O_4_/MWCNTs electrode materials in 3 M LiOH aqueous electrolyte.

Electrode Material	*R*_s_ (Ω)	*C*_dl_ (F)	*R*_ct_ (Ω)	*Z*_w_ (Ω s^−1/2^)	*τ* (s rad^−1^)	(°)
Mn_3_O_4_	0.36	3.16 × 10^−3^	0.52	0.38	3.77 × 10^−3^	37
CuMn_2_O_4_	0.31	5.66 × 10^−3^	0.48	0.33	2.69 × 10^−3^	45
CuMn_2_O_4_/MWCNTs	0.25	7.87 × 10^−3^	0.35	0.30	1.63 × 10^−3^	71

**Table 4:** Comparison of specific capacitance values of various supercapacitor electrode materials, evaluated in LiOH and KOH aqueous electrolyte solutions.

Electrode Material	Electrolyte	*C*_sp_ (F g^−1^)	Reference
CuMn_2_O_4_/MWCNTs	3 M LiOH	1652.9	This work
LiMnPO_4_/rGO	1 M LiOH	464.5	[59]
Ni–P/NiCo_2_O_4_	0.7 M LiOH	1240	[60]
Cu_3_SbS_4_/Ni–5	1 M LiOH	835.2	[61]
NiCo_2_O_4_//MoO_2_-C	1 M LiOH	94.9	[62]
NiCo_2_O_4_/rGO	2 M KOH	777.1	[63]
NiCo_2_O_4_/CNTs	1 M KOH	220	[64]
MnCo_2_O_4_/Ag NPs	6 M KOH	942	[65]
CNTs/C/NiMoO_4_	2 M KOH	1037	[66]

## Data Availability

The data presented in this study are available on request from the corresponding author. The data are not publicly available due to privacy or ethical restrictions.

## Supplementary Materials

The following supporting information can be downloaded at: https://www.mdpi.com/article/10.3390/nano12193514/s1, Figure S1: EDS spectra of Mn_3_O_4_, CuMn_2_O_4_ and CuMn_2_O_4_/MWCNTs materials, with their corresponding elemental percentage composition tables; Figure S2: Graphs of current against square root of scan rate for CuMn_2_O_4_/MWCNTs electrode in 3 M KOH (a) and 3 M LiOH (b) electrolytes; Graphs of log current against log scan rate of CuMn_2_O_4_/MWCNTs electrode material in 3 M KOH (c) and 3 M LiOH (d) aqueous electrolytes; Figure S3: Histogram of the specific capacitance of CuMn_2_O_4_/MWCNTs electrode material in 3 M KOH and 3 M LiOH aqueous electrolytes at various scan rates (a); CV curves of CuMn_2_O_4_/MWCNTs electrode material before and after 6000 cycles at 100 mV s^−1^ in 3 M LiOH aqueous electrolyte (b); CV curves of the bare MWCNTs electrode at 20 mV s^-1^ in both 3 M KOH and 3 M LiOH aqueous electrolytes (c); Figure S4: CV curves of Mn_3_O_4_ material at various scan rates in 3 M KOH aqueous electrolyte (a) and in 3 M LiOH aqueous electrolyte (b). Graphs of corresponding specific capacitances at various scan rates in 3 M KOH aqueous electrolyte (c) and in 3 M LiOH (d); Figure S5: GCD curves of Mn_3_O_4_ material at various current loadings in 3 M KOH aqueous electrolyte (a) and its corresponding graph of specific capacitances at various current loadings (b); GCD curves of Mn_3_O_4_ in 3 M LiOH aqueous electrolyte (c) with the respective graph of specific capacitances at different current loadings (d); Figure S6: Nyquist (a) and Bode (b) plots of the bare MWCNTs electrode in both 3 M KOH and 3 M LiOH aqueous electrolytes; Comparative Bode plot of CuMn_2_O_4_/MWCNTs electrode material in both 3 M KOH and 3 M LiOH aqueous electrolytes (c); GCD curves of the bare MWCNTs electrode, obtained at 2 A g^−1^, in both 3 M KOH and 3 M LiOH aqueous electrolytes (d); Table S1: EIS fitted data of the bare acid treated MWCNTs material in 3 M KOH and 3 M LiOH aqueous electrolytes. References [43,48,69,70,71,72] are cited in the Supplementary Materials.
