# Supplementary material for: Pseudocapacitive Effects of Multi-Walled Carbon Nanotubes-Functionalised Spinel Copper Manganese Oxide
Source: Nanomaterials (Basel). 2022 Oct 8;12(19):3514. doi: 10.3390/nano12193514 (PMC9565235; doi:10.3390/nano12193514)
Supplement: Supplementary file 1 [file nanomaterials-12-03514-s001.zip › nanomaterials-1820845-supplementary_updated.pdf]

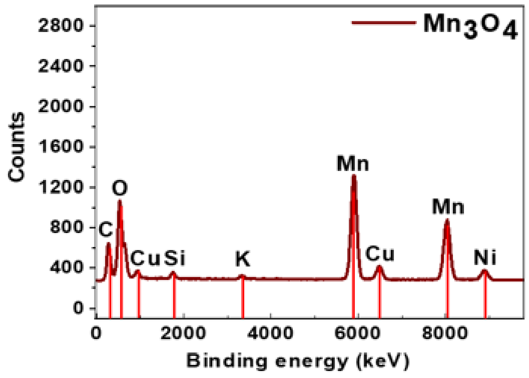

Element	Atomic %
C	21.03
O	50
Si	1.97
K	1.35
Mn	24.16
Cu	1.01
Ni	0.48
Total	100

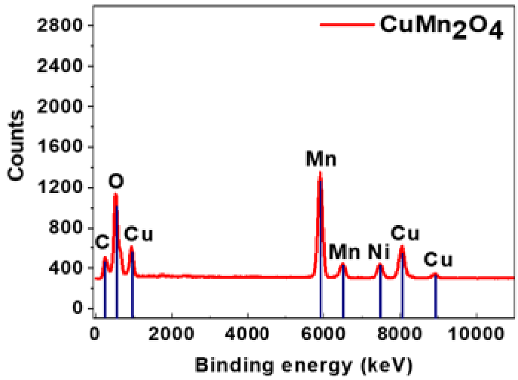

Element	Atomic %
C	18.07
O	34.29
Cu	19.78
Mn	26.62
Ni	1.24
Total	100

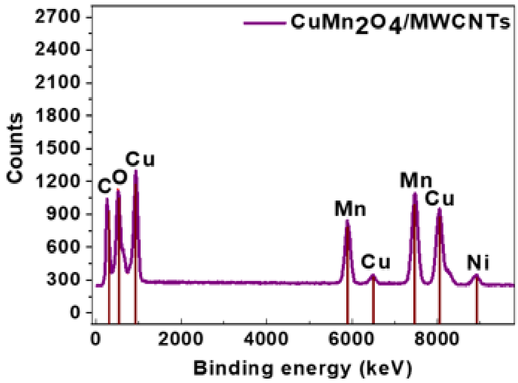

Element	Atomic %
C	22.01
O	23.34
Cu	36.33
Mn	16.96
Ni	1.36
Total	100

Figure S1. EDS spectra of Mn_3O_4, $CuMn_2O_4$ and $CuMn_2O_4$/MWCNTs materials, with their corresponding elemental percentage composition tables.

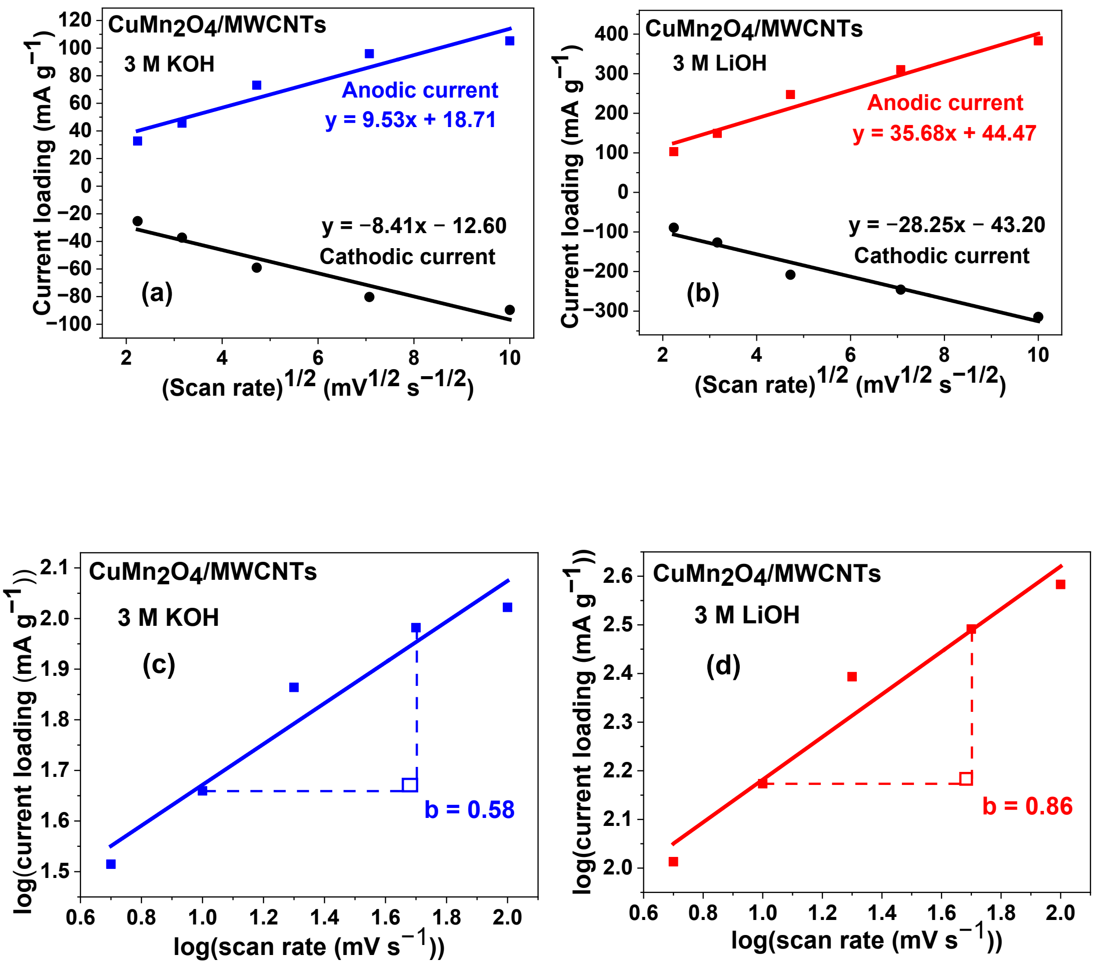

Figure S2. Graphs of current against square root scan rate for $CuMn_2O_4$/MWCNTs electrode in 3 M KOH **(a)** and 3 M LiOH **(b)** electrolytes; Graphs of log current against log scan rate of $CuMn_2O_4$/MWCNTs electrode material in 3 M KOH **(c)** and 3 M LiOH **(d)** aqueous electrolytes.

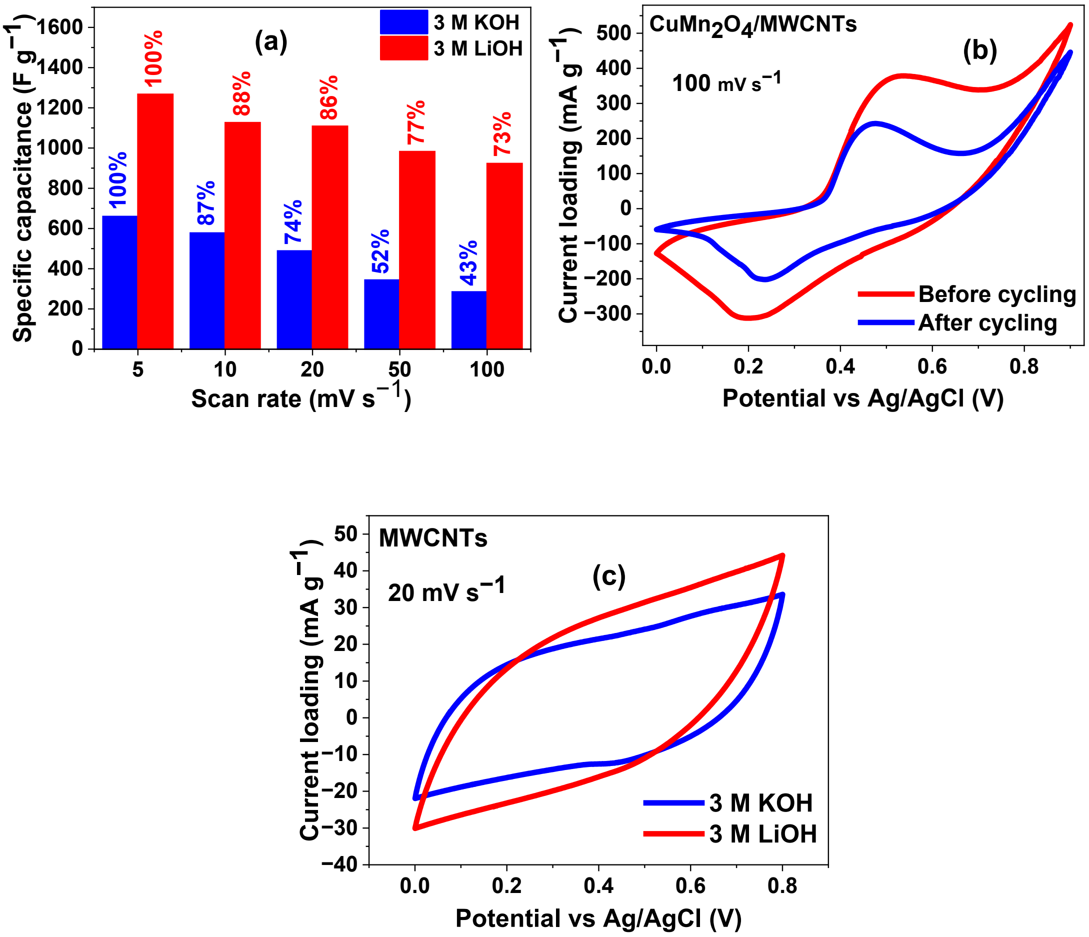

Figure S3. Histogram of the specific capacitance of $CuMn_2O_4$/MWCNTs electrode material in 3 M KOH and 3 M LiOH aqueous electrolytes at various scan rates **(a)**; CV curves of $CuMn_2O_4$/MWCNTs electrode material before and after 6000 cycles at 100 mV s^{-1} in 3 M LiOH aqueous electrolyte **(b)**; CV curves of the bare MWCNTs electrode at 20 mV s^{-1} in both 3 M KOH and 3 M LiOH aqueous electrolytes (c).

The CV curves of Mn_3O_4 at various scan rates and GCD data are presented in **Figs. S4 and S5**, respectively. The voltammograms in both 3 M KOH and 3 M LiOH electrolytes in **Fig. S4** (**a-b**) show a linear relationship between scan rate and peak current, with the material behaving reversibly at lower scan rates and irreversibly at higher scan rates thus indicating a quasi-reversible electrochemical system. The well-defined redox peaks can be ascribed to the redox transitions of Mn between the Mn^{2+}/Mn^{3+} and Mn^{3+}/Mn^{4+} redox couples in an alkaline medium. The specific capacitance was seen to decrease with scan rate as shown in **Fig S4** (**c-d**). This is due to the fact that at lower scan rates, more electroactive sites are accessible by the electrolyte ion whereas at higher scan rates, the penetration distance of the electrolyte ion into the material decreases and the ions are only limited to the surface. For the Mn_3O_4 electrode

material, a specific capacitance value of 108.23 F g^{-1} was recorded in 3 M KOH electrolyte at 5 mV s^{-1} whereas in 3 M LiOH electrolyte and at the same current loading, the specific capacitance obtained was 631.71 F g^{-1}. This is attributed to the easy intercalation/de-intercalation of Li^+ ions into the electrode [43,48,69–72].

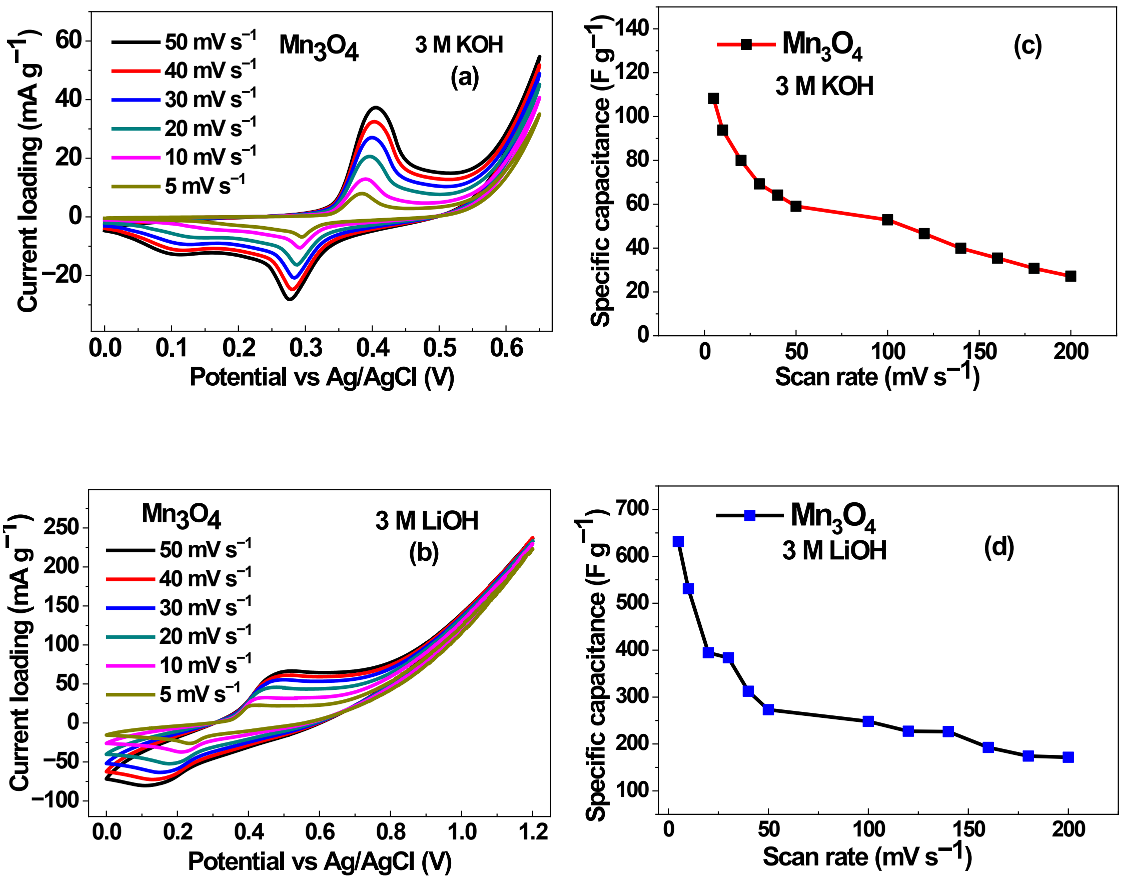

Figure S4. CV curves of Mn_3O_4 material at various scan rates in 3 M KOH aqueous electrolyte **(a)** and in 3 M LiOH aqueous electrolyte **(b).** Graphs of corresponding specific capacitances at various scan rates in 3 M KOH aqueous electrolyte **(c)** and in 3 M LiOH **(d)**.

The GCD curves of Mn_3O_4 at various current loadings are shown in **Fig. S5 (a-d)** with the electrode material exhibiting a longer discharge time at 450.86 s in LiOH electrolyte compared to the 79.69 s recorded in KOH electrolyte. Similarly, a specific capacitance of 853.15 F g^{-1} evaluated at a current loading of 0.5 A g^{-1} was measured in 3 M LiOH electrolyte whereas in 3 M KOH, 56.58 F g^{-1} was calculated at 0.5 A g^{-1} current loading. Generally, the specific capacitance was observed to decrease at higher current loadings as fewer electroactive sites are

available to the electrolyte ions due to sluggish kinetics at higher current loadings and scan rates.

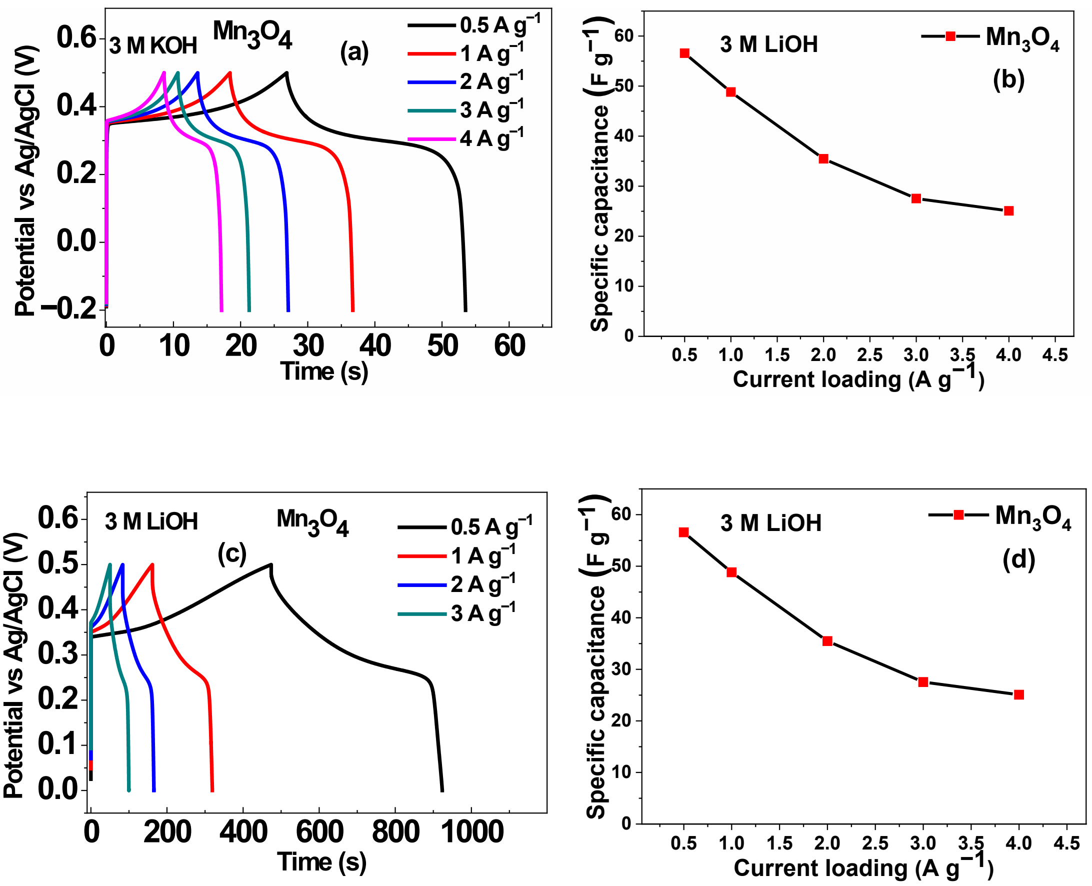

Figure S5. GCD curves of Mn_3O_4 material at various current loadings in 3 M KOH aqueous electrolyte **(a)** and its corresponding graph of specific capacitances at various current loadings **(b)**; GCD curves of Mn_3O_4 in 3 M LiOH aqueous electrolyte **(c)** with the respective graph of specific capacitances at different current loadings **(d)**.

The Nyquist and Bode impedance plots of the bare MWCNTs electrode, shown as supporting information in **Fig. S6**(a) and (b), illustrates a lower charge transfer resistance and higher phase angle in LiOH compared to KOH aqueous electrolyte. This complements the results obtained in CV of the bare MWCNTs electrode. Comparative Bode plots between the electrode in KOH and LiOH is presented in the supporting information as **Fig. S6**(c) with the $CuMn_2O_4$/MWCNTs in LiOH being more pseudocapacitive because of the associated higher phase angle. The values of

the kinetic parameters from EIS for the bare MWCNTs electrode in both KOH and LiOH electrolytes are summarised in **Table S1**.

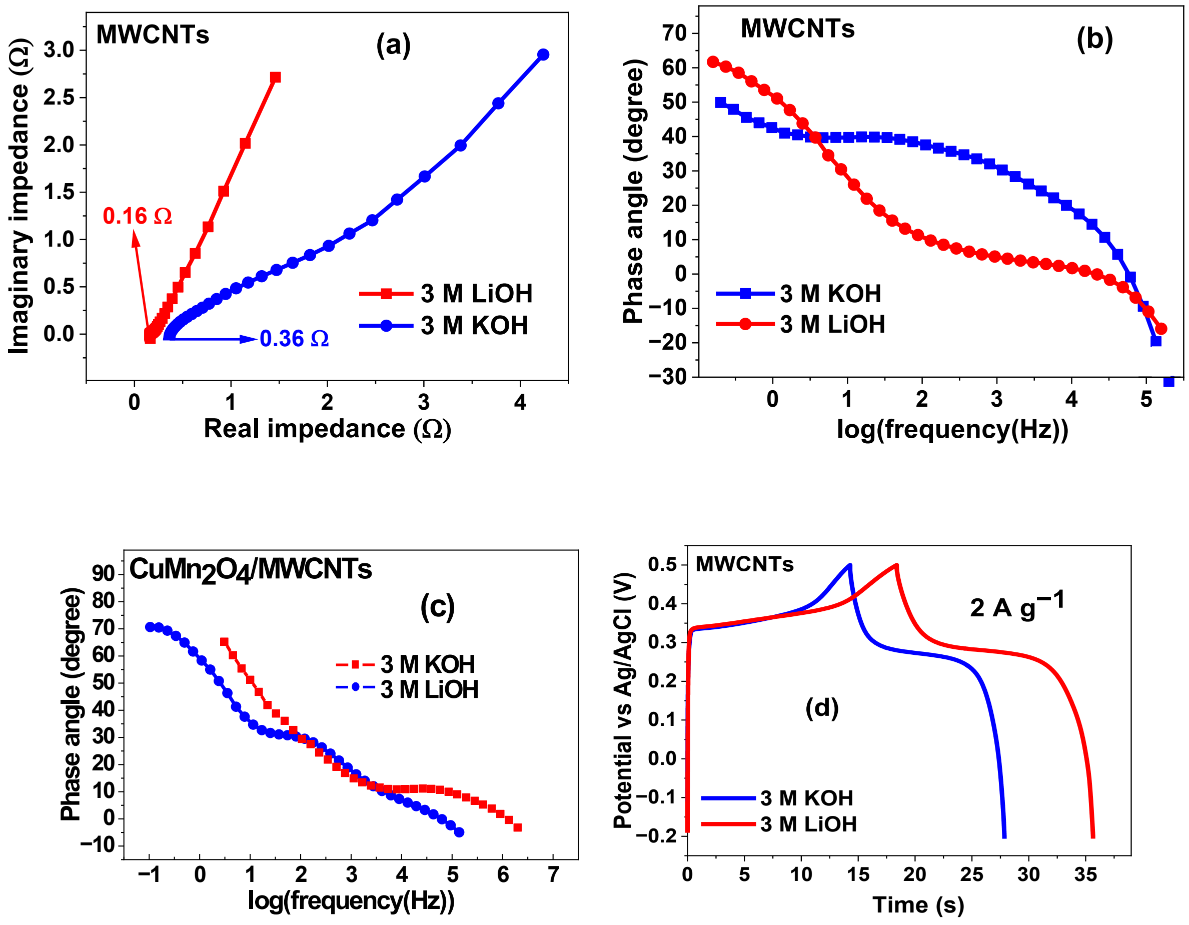

Figure S6. Nyquist **(a)** and Bode **(b)** plots of the bare MWCNTs electrode in both 3 M KOH and 3 M LiOH aqueous electrolytes; Comparative Bode plot of $CuMn_2O_4$/MWCNTs electrode material in both 3 M KOH and 3 M LiOH aqueous electrolytes **(c)**; GCD curves of the bare MWCNTs electrode, obtained at 2 A g^{-1}, in both 3 M KOH and 3 M LiOH aqueous electrolytes (d).

Table S1. EIS fitted data of the bare acid treated MWCNTs material in 3 M KOH and 3 M LiOH aqueous electrolytes.

**Electrode material**	R_s **(Ω)**	C_{dl} **(F)**	R_{ct} **(Ω)**	Z_w **(Ω $s^{-1/2}$)**	τ **(s rad^{-1})**	**(°)**

MWCNTs in 3 M KOH	0.85	2.47×10^{-3}	0.75	2.89	8.51×10^{-3}	50
MWCNTs in 3 M LiOH	0.38	4.13×10^{-3}	0.53	5.69	2.19×10^{-3}	61

GCD curves of the bare MWCNTs, shown as supplementary information in **Fig. S6**, illustrates a higher specific capacitance performance in LiOH (50.37 F g^{-1}) as compared to KOH (38.66 F g^{-1}).

References

43. I.I. Misnon, R.A. Aziz, N.K.M. Zain, B. Vidhyadharan, S.G. Krishnan, R. Jose, High performance MnO_2 nanoflower electrode and the relationship between solvated ion size and specific capacitance in highly conductive electrolytes, Mater. Res. Bull. 57 (2014) 221-230.

48. C. Zhong, Y. Deng, W. Hu, J. Qiao, L. Zhang, J. Zhang, A review of electrolyte materials and compositions for electrochemical supercapacitors, Chem. Soc. Rev. 44 (2015) 7484-7539.

69. A. Yuan, Q. Zhang, A novel hybrid manganese dioxide/activated carbon supercapacitor using lithium hydroxide electrolyte, Electrochem. Commun. 8 (7) (2006) 1173-1178.

70. M. Manickam, P. Singh, T.B. Issa, S. Thurgate, R.D. Marco, Lithium insertion into manganese dioxide electrode in MnO_2/Zn aqueous battery: Part I. A preliminary study, J. Power Sources 130 (2) (2004) 254-259.

71. A.I. Inamdar, Y.S. Kim, S.M. Pawar, J.H. Kim, H. Im, H. Kim, Chemically grown, porous, nickel oxide thin-film for electrochemical supercapacitors, J. Power Sources 196 (4) 2393-2397.

72. Y. Gu, J. Wu, X. Wang, W. Liu, S. Yan, Producing "Symbiotic" Reduced Graphene Oxide/Mn_3O_4 Nanocomposites Directly from converting Graphite for High-Performance Supercapacitor Electrodes, ACS Omega 5 (2020) 18975-18986.
